# Improving efficiency of fitting Cox proportional hazards models for time-to-event outcomes in genome-wide association studies (GWAS)

**DOI:** 10.1093/bioadv/vbad148

**Published:** 2023-10-13

**Authors:** Val Gebski, S Sandun M Silva, Karen Byth, Alicia Jenkins, Anthony Keech

**Affiliations:** NHMRC Clinical Trials Centre, University of Sydney, Camperdown, NSW 1450, Australia; NHMRC Clinical Trials Centre, University of Sydney, Camperdown, NSW 1450, Australia; NHMRC Clinical Trials Centre, University of Sydney, Camperdown, NSW 1450, Australia; NHMRC Clinical Trials Centre, University of Sydney, Camperdown, NSW 1450, Australia; NHMRC Clinical Trials Centre, University of Sydney, Camperdown, NSW 1450, Australia

## Abstract

**Summary:**

Technologies identifying single nucleotide polymorphisms (*SNPs*) in DNA sequencing yield an avalanche of data requiring analysis and interpretation. Standard methods may require many weeks of processing time. The use of statistical methods requiring data sorting, matrix inversions of a high-dimension and replication in subsets of the data on multiple outcomes exacerbate these times.

A method which reduces the computational time in problems with time-to-event outcomes and hundreds of thousands/millions of *SNPs* using Cox–Snell residuals after fitting the Cox proportional hazards model (*PH*) to a fixed set of concomitant variables is proposed. This yields coefficients for SNP effect from a Cox–Snell adjusted Poisson model and shows a high concordance to the adjusted *PH* model.

The method is illustrated with a sample of 10 000 *SNPs* from a genome-wide association study in a diabetic population. The gain in processing efficiency using the proposed method based on Poisson modelling can be as high as 62%. This could result in saving of over three weeks processing time if 5 million *SNPs* require analysis. The method involves only a single predictor variable (SNP), offering a simpler, computationally more stable approach to examining and identifying SNP patterns associated with the outcome(s) allowing for a faster development of genetic signatures. Use of deviance residuals from the *PH* model to screen *SNPs* demonstrates a large discordance rate at a 0.2% threshold of concordance. This rate is 15 times larger than that based on the Cox–Snell residuals from the Cox–Snell adjusted Poisson model.

**Availability and implementation:**

The method is simple to implement as the procedures are available in most statistical packges. The approach involves obtaining Cox-Snell residuals from a *PH* model, to a binary time-to-event outcome, for factors which need to be common when assessing each *SNP.* Each *SNP* is then fitted as a predictor to the outcome of interest using a Poisson model with the Cox-Snell as the exposure variable.

## 1 Introduction

New technologies have helped identify genetic differences between diseased and nondiseased populations and/or absence/presence of risk factors for disease. However, the volume of data provided by such technologies can occupy several thousand terabytes of storage space. The most common type of genetic variation among individuals are single nucleotide polymorphisms, labelled *SNPs* (pronounced “snips”).

Complex microarrays interrogate the genomic sequence with the potential to identify tens to hundreds of million *SNPs* (LaFramboise 2009). The sheer volume of such data presents challenges often requiring development of novel methods of analysis. Standard statistical methods can be sensibly applied to the enormous number of *SNPs* evaluated based on clinical studies having sample sizes ranging from a few hundred subjects (Sharafeldin *et al.* 2020) to tens of thousands (Polack *et al.* 2020). Due to both the cost and resources required to identify *SNPs*, measuring the *SNP* association with many different endpoints in a discovery framework might be included in the experimental plan. Since the aim of a *SNP* analysis is to identify whether an individual *SNP* is associated with the outcome of interest, the focus is on the strength of the association rather than on the numerical value of any estimated measure of association. The *SNP P*-value from the model provides an indication of the strength of association between *SNP* and outcome. For microarray discovery studies a *P*-value of .001 has been suggested as being adequate (Simon *et al.* 2003). Since we are screening for potentially noninformative *SNPs*, the choice of threshold is not as critical here as in situations involving formal statistical inference. This approach is considered as a s*ingle-pass*.

In many instances, an *s-fold cross-validation* is also desirable to provide support for the validity of potential promising sets of *SNPs*. The observation that certain *SNPs* maintain strong association with outcome in random subsets of size n*[1−(1/*s*)] of the data, *n* being the sample size of the study helps to strengthen conclusions regarding the importance of these *SNPs*.

*S-fold cross-validation* is an alternative approach to the single pass for identifying promising *SNPs*.

The process of *s-fold cross-validation* is to randomly divide the data into *s* (generally) equal groups, *g*_1,_*g*_2_, …, *g_s_* each of size *n(g*_*i*_) *i *=* *1, …, *s*, with *s *=* *5 being generally sufficient. The single-pass approach is performed for each subset of cases of size *n-n(g_i_*,) (i.e. omitting group, *g_i_*) *i *=* *1, …, *s*. Promising *SNPs* for further evaluation are identified as those where the association with the outcome demonstrates a *P*-value below a predetermined threshold (say) in, say, at least 80% of the subgroups.

Finally, for many genome-wide association study (*GWAS*) discovery studies, which homozygous pair is the normal and which the mutation is unknown for individual *SNPs*. For example, if pairs of interest are AA and GG, we may not know whether having AA represents normal or a mutation. The whole process of either *single-pass* or *s-fold validation* needs to be repeated using the reverse code (i.e. in this example we reassign the pair of interest from AA versus not AA to GG versus not GG), adding to the resource and processing time burden.

For time-to-event outcomes (such as time-to-death or time to disease progression), a common approach is to fit the Cox proportional hazards (*PH*) model (Collett 2013). Fitting of *PH* models however relies on iterative methods requiring matrix inversions and data sorting. When the number of common baseline variables is large (>20) together with and the number of *SNPs* to be processed being in the order of hundreds of thousands, this model fitting requires extensive computational resources (memory and computational speed) (Selmer 1990) resulting in substantial computational time. To illustrate, suppose that a processing time of 0.5 s to fit a single *PH* model having 25 predictor variables (including the *SNP* of interest) to a dataset of 2000 cases is considered acceptable. A discovery experiment comprising 30 000 000 *SNP*s would require 174 days per outcome without allowing for any cross-validation. If nine outcomes were being investigated, 4.3-processing years would be required to produce meaningful results using the conventional *PH* statistical fitting methods.

The efficiency of the fitting procedures can be examined by considering ways of reducing the computational burden without greatly compromising the precision of the estimates. One approach is to draw on the well-established principle in statistical modelling between data and a statistical model: *data = fit + residual* (Hoaglin *et al.* 1991), assuming that the *fit* component accounts for most the signal of interest. If the *fit* does not account for all the signal in the data, the excess signal would be contained in the *residual*. Many formulations of residuals from a *PH* model have been proposed (Collett 2013) primarily in the context of goodness of model fit, the most popular being martingale, deviance and Schoenfeld. Martingale and deviance residuals are transformations of the Cox–Snell (*CS*) residual (Cox and Snell 1968). *CS* residuals have been used successfully to evaluate the goodness of fit of *PH* models in clinical settings (Kay 1977). As the *CS* residual has desirable distributional properties, a straightforward separation of any signal/association provided by the effect of *SNPs* which may be contained in the *CS residual* after fitting *k* predictor variables can be investigated. This approach is appropriate despite not being optimal for assessing model fit.

A second choice is to investigate the use of deviance residuals which have the advantage of being more symmetric that martingale residuals and can be approximated by a normal distribution (Vieland *et al.* 2020). This has the potential to allow for rapid identification of *SNP* associations using *z*-tests which, by not requiring any iterative methods, would result in increased time efficiency and being less resource intensive than the *PH* or that based on *CS* residuals. These gains however may come at the expense of poorer agreement with the *PH* model (the gold standard), particularly when the proportion of censoring is high.

In the context of goodness of fit of a *PH* model (Crowley and Hu 1977) suggested adjusting the *CS* residuals by adding a constant [either unity or *ln*(2)] to the censored observations to account for the fact that the actual time to event is unknown. However, with the potential volume of data under consideration in most GWAS studies, it would be time prohibitive to examine the *PH* assumption for each individual *SNP* and it is not unreasonable to assume that the *PH* model is broadly applicable to all *SNPs*. The *PH* assumption should however be tested on the *k* predictor variables which are common to each model evaluating the individual *SNP*. Schoenfeld residuals (Schoenfeld 1982, Harrell and Lee 1986) can be used to screen out predictors violating the *PH* assumption to ensure that using the *CS* residuals is appropriate and that inferences regarding individual *SNPs* are based on sound methods.

The present study is focused on adapting established methodologies and Poisson regression models to improve the processing time required to identify promising *SNP* associations with time-to-event outcomes in clinical medicine.

### 1.1 Motivating example

The FIELD trial (The *FIELD* study Investigators 2005) was a large randomized controlled trial in 9795 diabetic patients investigating the extent to which fenofibrate could reduce the risk of either nonfatal myocardial infarction or death from coronary heart disease (primary outcome). Blood samples were collected during the study. A genomic study targeting some 37 million *SNPs* was performed on 5237 patient samples, this being the largest study of its type in a diabetic population. Nine time-to-event endpoints are of key interest, these being time to: coronary heart disease mortality (primary outcome); nonfatal myocardial infarction (MI); requirement for laser therapy (retinopathy); amputation; stroke; nonhemorrhagic stroke; revascularization (by-pass); coronary revascularization and cardiovascular and total mortality. It was deemed necessary to include 37 clinical, disease and patient predictor variables in all the *SNP* analyses. The inclusion of these predictors was guided by (i) whether individual predictors were significantly associated with the outcome(s) in this study; (ii) clinical/external knowledge of their association with the outcomes of interest; and (iii) whether similar GWAS studies conducted globally identified certain predictors and the pooling of all the results would strengthen the genetic interpretation relating to individual *SNPS*. The statistical method for the analysis involves fitting the same 37 predictor variables with each *SNP* in turn as a binary predictor (coded for homozygous for one genotype and heterozygous for the rest) to each outcome variable using a Cox proportional hazards regression (*PH*) model. The use of *PH* models rather than logistic regression models for time to event outcomes (whether or not a subject has experienced an event) is known to have more statistical power to identify associations with these outcomes if they truly exist (van der Net *et al.* 2008, Hughey *et al.* 2019).

While much attention has been given to developing methods aimed at analyzing high-dimensional genomic data, there is also interest in methods which identify promising *SNPs* where computational burden is not of major concern (Bermingham *et al.* 2015). In our study, the routine statistical analyses are resource intensive. We sought computationally faster alternative ways of identifying (higher dimensional) genomic profiles based on the results of analyses of individual *SNPs*.

As a crude estimate of the time required to analyze this data, assume the processing time for each individual *SNP* together with 37 predictor variables fitted in a Cox *PH* model is ∼0.5 s. An analysis of 5 million *SNPs* would take about one processing-month per outcome and over 10-processing months needed to analyze all 9 outcomes. If a 5-fold cross-validation approach was used (see below), this would require ∼5 processing-months per outcome and at least 45 months for all nine outcomes. Identifying which homozygous combinations carry the association signal would double these estimated times.

## 2 Methods

*Overview:* To improve computational efficiency, we propose a two-stage approach. In the first stage, a *PH* model is fitted to the time-to-event outcome with predictors being just the variables which will be common to all each *SNPs*. The residuals from the predictor estimates in model are also obtained. For the *FIELD* example discussed above, this would comprise the 37 predictor variables and the corresponding residuals from the fitted model. In the second stage, S*NP* categories (homozygous/heterozygous) together with these residuals are fitted to the dependent variable of event status (coded say, 1 for and event, 0 otherwise) to identify which *SNPs* have categories statistically associated with the risk of an event.

We describe aspects of the *PH* model in the context of fitting common predictor variables and *SNPs* together with their relationship to the Poisson model. The two-stage approach using the *PH* model and both *CS* and deviance residuals is developed and applied to obtain the computational times using the *FIELD* study comprising 10 000 *SNPs* as an experiment. These times are compared for all three approaches; the “full” *PH* model (all common predictor variables with each *SNP* in turn), and the two-stage approach based on *CS* and deviance residuals. Finally, we examine the concordance between the “full” *PH* model (gold standard), and the two-stage approach based on both *CS* and deviance residuals. Assuming the *PH* is a reasonable model choice, estimation of time-to-event evaluating large (>10^5^) *SNP* datasets and comprising many common predictor variables, the fitting separate *PH* models can impose a major computational burden due to the requirements for data sorting, matrix inversion and iterative numerical methods.

The novelty of the proposed method is to improve computational efficiency by separately fitting of the component comprising the common predictor variables to that of fitting each *SNP*. The *PH* model is fitted to the common components only once, and effect estimates of each *SNP* are obtained using the *CS* residuals in a Poisson model. This requires fewer iterations together with eliminating the need for data sorting and inversion of higher dimensional matrices. The approach could potentially be extended to estimate interaction effects between *SNPs*.

### 2.1 Method based on Cox–Snell residuals

#### 2.1.1 *PH* model

Assume *N SNPs* have been selected for screening. For each *SNP* in turn, a *PH* model with *k *+* *1 predictor variables (viz. the *SNP* and *k* required predictor variables) is fitted to the sample of size *n* cases.

The Cox *PH* model has the form $h\left(t\right)=$*h*_0_$\left(t\right)e^{\beta \prime X}\;$where for time *t, h_0_*(*t*) is the baseline hazard. ***β***′= (*β_1,_ β_2_*, … + *β_k,_ β_SNP(j)_)* is the (*k *+* *1) vector of model coefficients, ***Xβ*** = *β_1_ X_1_* + … + *β_k_ X_k_ + β_SNP(j)_ SNP*(*j*), where ***X*** is the [n* (*k + *1)] matrix of predictor variables with *SNP*(*j*), *j *=* *1, …, *N* being the *SNP* of interest.

The partial log-likelihood from the Cox *PH* model is:


$$l\left(\beta \right)=\sum_{i=1}^{n}\delta_{i}[\beta x_{i}-ln\left(\sum_{\mathrm{l\epsilon R}\left(t_{i}\right)}e^{\beta \prime x_{l}}\right)]\;$$

*δ*_j_ is the outcome event indicator with the value 1 if the subject has experienced the event of interest, and 0 otherwise; ***x_i_′***is the *i*th row vector of covariate values of interest for subject *i* (Collett 2013); *t_i_* is the follow-up time for subject *i*, and *R(t_i_)* is the set of all individuals who are event free and not censored before time *t_i_* (termed “risk-set”).

The maximum likelihood estimates are then obtained by forming the score equations ***U***(***β)*** from the partial derivatives of *l*(***β***) with respect to *β_u_*, *u *=* *1, …, *k *+* *1 and, estimates of ***β_u_***, ${\hat{\beta }}_{u}\;$ are obtained by solving ***U***(***β) = 0***.

The solutions are obtained through an iterative process using gradient methods of Newton-Raphson with the Marquart-Levenberg dampening to improve numerical stability (Nash 1990). The variances (and standard errors) of these estimates are then calculated from the inverse of the (*k *+* *1)* (*k *+* *1) information matrix of second derivatives. For each *SNP*, fitting the *PH* model typically requires 3–5 iterations each involving matrix inversion of the (*k *+* *1)*(*k *+* *1) gradient matrix.

#### 2.1.2 Poisson formulation for the Cox PH model

By drawing on the fact that, in the absence of tied observations, and assuming the *k* predictor variables are not time dependent, the maximum likelihood estimates from a Poisson model based on an augmented dataset and a *PH* model can be shown to be identical (Whitehead 1980). The dataset augmentation can be described as follows. Assume we have a dataset of *n* cases comprising *T* distinct ordered event times, *t_1_, t_2_*, *…*, *t_T_*; *t_1_ < t_2_* < *… <t_T_*, *T ≤ n.* (i)–(iv) below outlines the construction of the augmented dataset.

- Construct block 1 containing the case with the event at time *t_1_* having an event status 1 and the remaining observations with follow-up times > *t_1_* have their times set to *t_1_* and their event status set to 0, and the block indicator 1 added as an extra column to this dataset. Any censored observations with a follow-up time < *t_1_* do not contribute to the likelihood and can be excluded.
- Block 2 will comprise the case with the event at time *t_2_* having an event status 1 and the remaining observations with follow-up times > *t_2_* having their times set to *t_2_* and their event status set to 0 and the block indicator 2 added as an extra column.
- The procedure is repeated for the remaining times *t_3_*, …, *t_T_*. The blocks *1*, *2*, …, *T* are augmenting row-wise, and, additional columns comprising *T* indicator variables, where indicator *T_i_* =1 if the observations are located in block *i*, and 0 otherwise are created. This forms a dataset comprising *k* predictor columns, an event status column, and *T* block indicator columns.
- A Poisson regression is then fitted with outcome being the event status (*Y*), and predictor variables the original *k* predictors together with the *T* −* 1* indicators (*X’s*). The coefficients from fitting this model corresponding to the *k* predictor variables give estimates of the hazard ratio for each variable, and are identical to those which would be obtained from fitting a *PH* model directly. The intercept and remaining *T* −* 1* coefficients can be used to estimate the baseline hazard function at each of the *T* time points detailed in *2.4*.

Theoretical details of the relationship between these two approaches is given in Johansen and Johansen (1983) where he notes that in the Poisson regression formulation, estimates of the hazard ratios for the *k* predictor variables are obtained through fitting the additional *T* −* 1* (and theoretically, an infinite number) parameters. With medium to large number of observations, the augmented dataset can become huge which diminishes the practical usefulness of this approach.

### 2.2 Cox–Snell residuals

The intuitive formulation of *data = fit + residual* (i.e. as vertical distances from the fitted equation) not easily represented in the *PH* framework. To overcome this restriction, formulations involving the cumulative hazard and survival times can be used to gauge the appropriateness of *PH* models. An established mathematical fact is that the survival function *S(T)* for a continuous survival time *T* has a uniform distribution on the interval (0,1). Commonly, the cumulative hazard function at time *t* is estimated as ${\hat{H}}_{0}\left(t\right)=-\log(\hat{S}\left(t\right)),\;\hat{S}\left(t\right)\;$being the cumulative survival function estimated by the Kaplan–Meier method.

The method of transformations for functions of random variables, can be used to derive the probability density function of${\;\hat{H}}_{0}(t)$. The probability density random of the random variable *U*(*x*) where *X*=${\;\hat{H}}_{0}(t)$= –log(*S(t)*), can be written as:


$$\frac{{f_{U}\left(u\right)=f}_{T}\left\{H_{0}^{-1}\left(e^{-u}\right)\right\}}{\left|\frac{du}{dt}\right|};\;\frac{du}{dt}=\frac{f_{T}(t)}{S(t)}.$$

This expression can be shown to reduce to *f_U_(u)* = e^*−u*^, the probability density function of an exponential random variable U with unit mean (Collett 2013). This provides motivation for the construction of residuals having similar behavior to an exponential distribution with parameter 1.

For individual *i, i = 1*, *…*, *n*; the Cox–Snell residual is given as:


$${r(i)}_{CS}={\;\hat{H}}_{0}\left(t\right)e^{\hat{\beta }\prime x_{l}}$$

where$\;\hat{\beta }x_{i}$ is defined in Section 2.1.1 and ${\;\hat{H}}_{0}(t)$ the estimated cumulative baseline hazard. Provided the fitted *PH* model fits the data, then the *CS* residuals will follow a unit exponential distribution. This condition is generally satisfied in most *GWAS* studies.

In the current example, any *SNP* effect associated with the outcome would be contained within these residuals after fitting a *PH* model with just the *k* predictor variables. Since *CS* residuals are always positive, if *SNP*(*j*) *j = 1*, …, *N* is associated with the outcome, we use the relationship: *r(i)_CS_ = SNP*(*j*) *effect + (a further) residual* to isolate this *SNP* effect.

We note that *CS* “residuals” are a transformation of the estimated survival function and not deviations from a postulated model. Measuring how much the residuals deviate from a unit exponential is their main strength. The problem is now to tease out any *SNP* effect from the (right-censored) *CS* residual.

### 2.3 Fitting right-censored exponential data using Poisson modelling

Right-censored data having an exponential distribution can be modelled using a Poisson distribution by selecting an appropriate offset or exposure component (Aitkin and Clayton 1980). In our case, we fit a Poisson model with *Y* being the outcome variable (coded 0 if the observation is censored and 1 if an event has occurred), the predictor (*X*) being the *SNP* (coded homozygous or not) and the offset or exposure variable being the *CS* residual, *r(i)_CS_.* Using this offset, we effectively fit *ln*{*r(i)_CS_*} as an explanatory variable with fixed coefficient of 1. The (incidence density) parameter λ from the *CS adjusted* Poisson model where *ln(λ)* = ***β′x*** + *ln*{*r(i)_CS_*} approximates the hazard ratio associated with the *SNP* effect in a *PH* regression model fitting all *(k + 1)* variables.

### 2.4 Equivalence of the CS residuals between *PH* and Poisson formulations

Using the notation in Section 2.3, if the *k* predictor and *T* −* 1* time indicator variable are fitted using a Poisson regression model, and (omitting *T_1_*) if,


$$\hat{\beta }\prime x={\hat{\beta }}_{0}+{\hat{\beta }}_{1}x_{1}+..+{\hat{\beta }}_{k}x_{k}+{\hat{\beta }}_{k+2}I_{k+2}+{\hat{\beta }}_{k+2}I_{k+2}+..+{\hat{\beta }}_{k+T}I_{k+T},$$

then the predicted values for each observation from this model are given by $e^{\hat{\beta \prime }x}\;$(assuming no tied observations). The predicted values can be partitioned as


$$\{e^{{\hat{\beta }}_{1}x_{1}+..+{\hat{\beta }}_{k}x_{k}}\}\left\{e^{{\hat{\beta }}_{0}+{\hat{\beta }}_{k+1}I_{k+1}\;+\;\ldots \;+\;{\hat{\beta }}_{k+T}I_{k+T}}\right\},$$

the *k* coefficients ${\hat{\beta }}_{1},\ldots \;{\hat{\beta }}_{k}\;$ being identical to that obtained from the *PH* models while the remaining *T* coefficients ${\hat{\beta }}_{0},{\hat{\beta }}_{k+2}\ldots \;{\hat{\beta }}_{k+T}$contribute to the estimate the cumulative baseline hazard of${\;\hat{H}}_{0}(t)$.

The coefficients in second component correspond to the time indicators, and for time period *t_r_* the contribution of these terms to the estimate of the cumulative hazard is $e^{{\hat{\beta }}_{0}+{\hat{\beta }}_{k+r}\;},\;$*r = 2, T.*

$e^{{\hat{\beta }}_{0}\;}$ is the contribution in the interval [*t_1_, t_2_*); $e^{{\hat{\beta }}_{0}+{\hat{\beta }}_{k+2}\;}$ in the interval [*t_2_, t_3_*), …, $e^{{\hat{\beta }}_{0}+{\hat{\beta }}_{k+T}\;}$ in the interval [*t_T_*, ∞). As there are no events in the interval [0, *t_1_*), the estimate of the cumulative hazard is 0.

The cumulative baseline hazard${\;H}_{0}(t)$ over time is estimated from the partial sums of these individual quantities which are identical to that obtained from fitting the *PH* model directly. An example of these computations is given in the appendix. The connection between the *PH* and Poisson formulations with the *CS* residuals also being components from the Poisson formulation strengthening the use of this two-stage strategy of fitting the *k* predictors followed by the *SNP* to the *CS* residuals.

### 2.5 Deviance residuals

As mentioned in Section 2.2, CS residuals are transformations of the survival function and differ from residuals in the traditional sense of deviations from a postulated model. Several residuals from a *PH* model based on distributions which are more symmetrical have been developed, with the *deviance residual* being more commonly used. The attraction of the deviance residuals is that they behave more like residuals from a linear model with observations having large deviance residuals demonstrating a poor model fit. For the *PH* model, the *deviance residual r(i)_D_* for the *i*th observation is defined as a transformation of *r(i)_CS_* viz., if we let *r(i)_M_ = δ_i_ − r(i)_CS_*; *δ_i_ =* 1 if the *i*th observation is uncensored, 0 otherwise and s*ign*() taking the value

+1 if *r(i)_M_ >* 0 and −1 if r*(i)_M_ <* 0. (Therneau *et al.* 1990) then:


$${r(i)}_{D}=\mathrm{sign}\left[{r\left(i\right)}_{M}\right]\sqrt{-2{[r\left(i\right)}_{M}+\delta_{i}ln\{{r\left(i\right)}_{\mathrm{CS}}\}]}.$$

The distribution of the deviance residuals is more symmetrical than the *CS* residuals and is approximately normally distributed when the percentage of censored observations is <25%. Since *t*- and *z*-tests are robust to moderate departures from normality (Havlicek and Peterson 1974, Garren and Osborne 2021) this approach may offer an attractive efficient alternative to *CS adjusted* Poisson approach for screening the voluminous number of *SNPs* as, other than that used to obtain the deviance residual, no further iterative procedure is required (Yoo *et al.* 2001, Joshi *et al.* 2016).

Since *PH* regression models have more statistical power than logistic regression models (Hughey *et al.* 2019), the proposed approach should retain this advantage while being more computationally efficient. Thus, the problem of fitting (*k *+* *1) predictor variables requiring several (*k *+* *1)*(*k *+* *1) matrix inversions for each *SNP* is reduced to one of fitting a single predictor variable, the *SNP* (after first obtaining the *CS* residuals from the *k* common predictors). Although the Poisson fitting procedure is iterative, no matrix inversions are involved, and processing time should be substantially reduced. We note that the incidence density or risk ratio from the Poisson model is not identical to the HR from the Cox *PH* model. In the genetic discovery framework, enormous analyses involving millions of *SNPs*, some deviation from the *PH* model (gold standard) would be acceptable in lieu of large gains in computational and processing efficiency. We assume the *PH* model provides a reasonable approximation to the underlying association as does the Poisson model, and note that in the survival package (Therneau 2023) in R-Studio (R StudioTeam 2020), in which the analyses were performed, ${\;H}_{0}(t)$ is estimated from $-\log[\hat{S}\left(t\right)]$ using the Kaplan–Meier estimate for *S(t)*.

## 3 Experiments

We use the *FIELD* example discussed previously to compare the performance of the proposed approach with that of the *PH* model. The *GWAS* component was based on 5237 blood samples of which 4516 were eventually deemed to have yielded useful *SNP* information. For the comparison dataset we chose 10 000 *SNPs* from positions 60 000—70 000 with sufficient data to fit the *PH* models. For each *PH* model, the *SNP* and fixed subset of 37 required predictor variables were included giving a 4516 × 38 design matrix. The primary outcome considered of the study was either nonfatal myocardial infarction or death from coronary heart disease.

We fitted 10 000 *PH* models using each *SNP* in turn to obtain estimates of the coefficients (log hazard ratio) together with standard errors. Standardized coefficients (the *z*-value used to determine statistical significance in an inferential setting) were computed by dividing the estimated coefficient by its standard error. We next calculated the *CS* residuals, *r(i)_CS_*, *i *=* *1, …, 4516, from a single *PH* model containing only the subset of 37 required predictor variables. Using *r(i)_CS_* as the offset or exposure variable, for each *SNP*(*j*) *j = 1*, …, *N* a Poisson regression was fitted with mean


$$\ln\left(\hat{\mu }\right)=\beta_{0}+\beta_{1}S\mathrm{NP}\left(j\right)+\ln\{{r\left(i\right)}_{\mathrm{CS}}\};\;i=1,2,.,4516.$$

### 3.1 Assessing concordance between the *PH* fitting, *CS adjusted* Poisson, and deviance residuals

Summary timing statistics from these two analytical approaches together with differences of the standardized coefficients from the estimated models are shown in Table 1.

**Table 1.** Descriptive timing and model fitting statistics for the sample of 10 000 *SNPs*.^a^

Variable	Mean	SD	Min	Median	Max
Poisson*_csadj_*: processing time (s) per *SNP*					
Iterations	0.0115	0.0034	0.0051	0.0108	0.0519
Fitting model	0.0337	0.0098	0.0165	0.0321	0.2989
Cox *PH*: processing time (s) per *SNP*					
Sorting data	0.0003	0.0050	0.0001	0.0002	0..4998
Iterations	0.0229	0.0055	0.0137	0.0204	0.0998
Fitting model	0.0902	0.0877	0.0483	0.0712	1.1656
Deviance residuals: processing time (s) per *SNP*	0.0075	0.0020	0.0054	0.0068	0.0656
Standardized coefficients (*z*-value for *SNP*)					
Absolute difference: (Cox *PH −* Poisson*_csadj_*)	0.0141	0.0138	<10^−5^	0.0105	0.2254
Absolute difference: (Cox *PH −* deviance)	0.2311	0.2774	<10^−5^	0.1666	5.5796
Coefficient: ln(risk estimate)					
Absolute difference: (Cox *PH −* Poisson*_csadj_*)	0.0069	0.0108	2*10^−6^	0.0039	0.2412

a Poisson*_csadj_*, Poisson model adjusted for Cox–Snell residuals.

We see a reassuring consistency in the summary statistics from the two approaches. The mean absolute difference of the (standardized) coefficients from the univariable *CS adjusted* Poisson analyses (fitting each SNP in turn) from those estimated using the *PH* model is 0.0141. As the coefficients from both *PH* and the *CS adjusted* Poisson models estimate the logarithm of the relative risk (hazard for the *PH* model and incidence density for the Poisson) between the *SNP* categories these differences have a direct interpretation in terms of risk differences. Absolute differences between these coefficients provide a guide as to how well the Poisson approach approximates the *PH* model. Table 1 shows that the mean absolute difference is <0.007 (median being <0.004) suggesting not only strong agreement but also the closeness of the estimated coefficients from the two methods.

If deviance residuals are used, the difference between the means for the two *SNP* categories is a measure of *SNP* effect. This is the slope coefficient of the slope of a linear regression, the predictor being the *SNP* category and the response being the deviance residual. The average absolute deviation between the standardized slope coefficient (*z*-value) and the standardized coefficient from the *PH* model is 0.2311, with the average maximum deviation being 5.58. As these standardized coefficients are key in identifying potential associations with outcome, large deviations from the *PH* model would be a major cause for concern. While the deviance residual approach is very fast, 0.0075 s/*SNP*, average processing time, 91% and 78% faster than the *PH* and *CS adjusted* Poisson models, the accuracy of the results to the standardized *PH* coefficients may be a major drawback.

**Figure 1.**
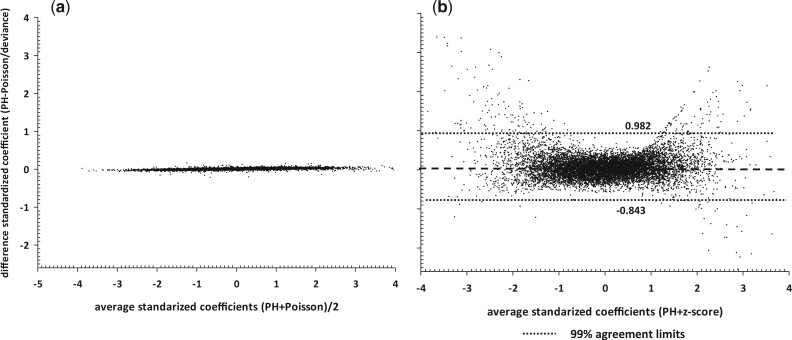
Bland–Altman plots: agreement between standardized SNP coefficients from *PH* model and (a) Poisson, (b) deviance residuals *z*-scores. Differences (*Y*-axis) are *PH—*Poisson, *PH—*deviance estimates.

Agreement between the estimates of the standardized coefficients (coefficient divided by its standard error) can be assessed using the Bland–Altman plot. The Bland–Altman plot (Oldham 1962, Bland and Altman 1986, 1999) is a graphical method for assessing agreement between two quantitative measurements obtained from two methods 1 and 2, by plotting the differences between the two measurements (Δ_1,2_) against their average. Various levels of limits of agreement can be constructed based on the standard deviation of the differences (*s*_diff_). Figure 1a shows the Bland–Altman plot of the *PH* and *CS adjusted* Poisson standardized coefficients for the 10 000 SNPs. The 99% limits of agreement are (−0.0512, 0.0503); 20 *SNP* differences being < −0.0512 and 76 *SNP* differences being >0.0503 (0.96% in total). The negligible bias ($\bar{\Delta }$ = −4.67*10^−4^) and tight agreement boundaries demonstrate the feasibility of reducing time and resources for screening large volume of *SNPs* using *CS* residuals. The Bland–Altman plot of the agreement between the *PH* and *z* value standardized coefficients for the 10 000 *SNPs* is shown in Fig. 1b. The overall bias (mean of the differences Δ_*i*_, between the standardized scores) is 0.07. The 99% Bland–Altman limits of agreement are (−0.843, 0.982) with 29 (0.3%) *SNP* differences being <−0.843 and 185 (1.85%) *SNP* differences being >0.982.

### 3.2 Assessing computation times between *PH, CS adjusted* Poisson models, and deviance residuals

Summary statistics of the processing time (in seconds) taken to fit the *PH* and *CS adjusted* Poisson models for each *SNP* are presented in Table 1. For the *PH* model the average time is 0.0902 s, being as long as 1.1656 s. The *CS adjusted* Poisson approach has on average, 63% shorter processing time (median, 55% shorter) with the longest time being 74% lower than the longest time for the *PH* model. To provide some idea of how much this computational component contributes to the total time, times to perform the iteration component (data sorting and matrix inversion) in the fitting of the *PH* and Poisson models are also shown. For the *PH* model, on average the sorting and matrix inversions contribute 26% of the total time, the balance being driven by the number of iterations taken and arrangement of the data/output. The iterative component for the *CS adjusted* Poisson calculations comprises 34% of the total time to fit the model. The iteration time to fit the *CS adjusted* Poisson model is 49% of the time taken for iteration and sorting component of the *PH* model. Linear regression of *SNP* categories on the deviance residuals has the fastest computational times.

The processing times per *SNP* for the *PH* and *CS adjusted* Poisson models and linear regression on the deviance residuals using *5-fold cross-validation* are shown in Table 2.

**Table 2.** Average processing time per *SNP* (in seconds) required using *5-fold cross validation* for 10 000 *SNPs* and 4516 cases.

		Cox *PH* (s)	SD	Poisson_*CSadj*_ (s)	SD	Deviance (s)	SD
Cross-validation number	1	0.0831	0.0369	0.0352	0.0248	0.0137	0.0057
	2	0.0830	0.0378	0.0350	0.0243	0.0135	0.0060
	3	0.0835	0.0403	0.0344	0.0231	0.0134	0.0051
	4	0.0820	0.0409	0.0349	0.0245	0.0134	0.0057
	5	0.0828	0.0431	0.0344	0.0229	0.0135	0.0051

Comparing processing times per *SNP* in Tables 1 and 2, compared to including all subjects, there is only a marginal benefit of 8%–9% shorter processing for each cross-validation analysis when using Cox *PH* and a 2%–4% increase with the *CS adjusted* Poisson approach. If *s-fold cross-validation* is used to identify promising *SNPs* (as is common), then the estimated processing time would be in the order of *s times* that required for single pass analysis using all subjects. To identify which homozygous pair carries the association with the outcome, all these analyses need to be repeated with the heterozygous *SNPs* grouped with the complementary homozygous alleles. This would double the total processing times. The performance of linear regression modelling on the deviance residuals is impressive, consistently 84% faster than the *PH* fitting and 60% faster than the *CS adjusted* Poisson approach.

Processing times for various stages in the Cox *PH*, *CS adjusted* Poisson analyses and linear regression on deviance residuals on the *FIELD* 10 000 *SNP* example for a single time to event outcome are shown in Table 3 together with an extrapolation of the results to an analysis of five million *SNPs*. The times are those taken on a single desktop system.

**Table 3.** FIELD discovery GWAS example: processing time (min) for a single time to event outcome for 10 000 *SNPs* based of a single pass or 5-fold cross-validation.

	Cox *PH*	Poisson	Deviance
Using a single processor			
First: homozygous pair single pass	15.04	5.61	1.25
5-Fold validation	54.45	22.78	8.84
Second: homozygous pair single pass	17.31	6.82	2.03
5-Fold validation	62.32^a^	27.69^a^	14.36^a^
Total time: single pass	32.35	12.43	3.28
5-fold validation	116.77	50.47	23.47
Extrapolating to 5 million *SNPs*: expected processing time (days)			
First: homozygous pair single pass	5.22	1.94	0.43
5-Fold validation	18.91	7.91	3.07
Second: homozygous pair single pass	6.01	2.37	0.71
5-Fold validation	21.63	9.61	4.99
Total time: single pass	7.03	4.32	1.14
5-Fold validation	45.70	17.72	8.15

a The 5-fold validation the times second pairs were approximated using the ratio of the times in the first pair, e.g. 62.32 = 17.31*(54.45/15.04).

The total time taken is obtained by running the models in a single session whereas times for the first and second homozygous pairs are recorded as two separate sessions. For the Cox *PH* model this is 15.04 min (single pass) and 32.35 min for two passes as opposed to 5.61 and 12.43 min respectively for the *CS adjusted* Poisson approach. The efficiency of the Poisson model over the *PH* for a two-pass approach is 62% faster and 57% faster for a 5-fold cross-validation. When the number of *SNPs* increases to 5 million, the 5-fold validation can result in a saving of at least 28 days processing time per outcome. As expected, the regression on deviance residuals demonstrates the fastest computational time, requiring approximately 8.5 days to process 5 million *SNPs* through a 5-fold validation process as opposed to 46 days for a *PH* model or 18 days for the *CS adjusted* Poisson.

### 3.3 Comparing the inferences based on the *PH* model and *CS adjusted* Poisson approach

Processing large volumes of data, we have seen that the *CS adjusted* Poisson approach and linear regression on the deviance residuals provides substantial improvement in processing efficiency, particularly when many outcomes are being considered and an s-fold cross validation is used in the screening process. We set a threshold *P*-value from the model to determine which *SNPs* demonstrate potential association with a particular outcome. As mentioned previously, a threshold of 0.001 has been suggested as being reasonable in a discovery context to identify candidate *SNPs* for further investigation.

The cross-tabulations between the *PH* and *CS adjusted* Poisson models for *P*-value thresholds of 0.001 and 0.002 are displayed in Table 4. With a 0.001 threshold, there were no discordant *SNP* results. With a 0.002 threshold, there were 7 (0.07%) discordant *SNP* results for which the maximum absolute difference between the *P*-values was 0.0004.

**Table 4.** Concordance of *SNP P*-values from *PH* and *CS adjusted* Poisson models (a) discordant pairs for a threshold of 0.001, (b) discordant pairs for a threshold of 0.002.

		(a) *PH* model			(b) *PH* model			
		≤0.001	>0.001	Total		≤0.002	>0.002	Total
Poisson	≤0.001	26		26	≤0.002	42	1	43
model	>0.001		9974	9974	>0.002	6	9951	9957
	Total	26	9974	10 000		48	9952	10 000

Discordant pairs: *P*-values							
	Threshold						
*P*-values		≤0.002					>0.002
*PH* model	0.0020	0.0019	0.0019	0.0017	0.0019	0.0019	0.0021
Poisson model	0.0024	0.0023	0.0021	0.0021	0.0021	0.0023	0.0020
Difference	0.0004	0.0004	0.0002	0.0004	0.0002	0.0004	−0.0001

Similarly, cross-tabulations for the concordance for the 10 000 *SNPs* between the *PH* and deviance residual *z*-test *P*-values using threshold levels of 0.002 or 0.001 is shown in Table 5.

**Table 5.** Concordance of *SNP P*-values from *PH* and *z*-test (deviance residuals) (a) discordant pairs for a threshold of 0.001, (b) discordant pairs for a threshold of 0.002.

		(a) *PH* model			(b) *PH* model			
		≤0.001	>0.001	Total		≤0.002	>0.002	Total
*z*-test	≤0.001	3	58	61	≤0.002	13	73	86
	>0.001	23	9916	9974	>0.002	35	9879	9952
	Total	26	9974	10 000		48	9952	10 000

At the nominal 0.001 and 0.002 thresholds there is substantially more discordance between *PH* and deviance residual *z*-test *P*-values than that between *PH* and *CS adjusted* Poisson model *P*-values. 81 and 108 discordant pairs in Table 5 compared to 0 and 7 discordant pairs in Table 4. The maximum absolute difference between *P*-values for the discordant pairs in Table 5 is 0.5108 (0.1% level) and 0.5196 (0.2% level).

**Figure 2.**
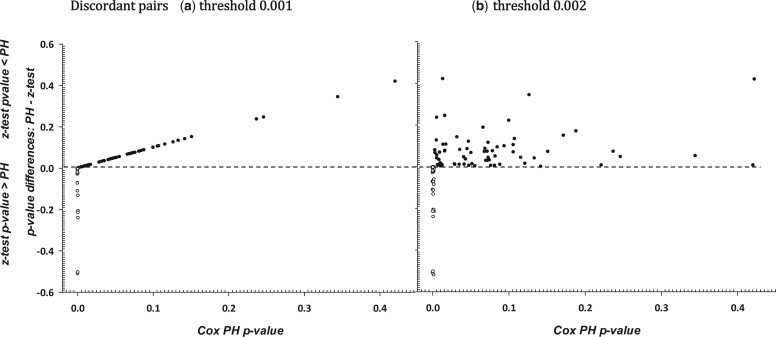
For the discordant pairs: *PH*_*p*-value_ − z_*p*-value_: Negative values, ○, where [z_*p*-value_ is  ≤  *α*, and *PH*_*p*-value_ >  *α*]; positive values, ●, where [z_*p*-value_ is  >  *α* and *PH*_*p*-value_ ≤  *α*]; (a) *α* = 0.001; (b) *α* = 0.002.

Due to the large number of discordant pairs, for these pairs, the differences *PH*_*p*-value_ − z_*P*-value_ are displayed against the *PH*_*p*-value_. These are shown in Fig. 2a for the threshold 0.001 and Fig. 2b for the 0.002 threshold. In Fig. 2a, positive differences indicate a small z_*p*-value_ (≤0.001) when the *PH*_*p*-value_ are large (.>0.001) with the reverse for negative differences. Larger *PH*_p-value_ are associated with increased bias. For the 0.002 threshold, no clear pattern in the differences is evident (Fig. 2b). Negative differences small *PH*_p-value_ (≤threshold) are associate with large z_*p*-value_ (>threshold).

The average negative differences are −0.096 and −0.102 for the 0.001 and 0.002 thresholds respectively. Further work is needed to determine the reasons behind the magnitude of the discordancy between the *PH* model and the two-stage approach based on the deviance residuals.

**Figure 3.**
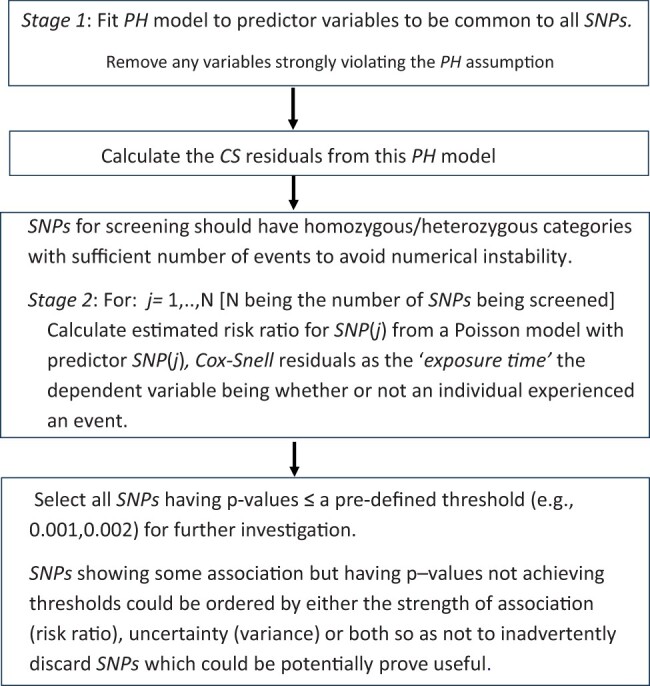
Steps in the two-stage process for using Cox–Snell residuals to identify *SNPs* having a strong association with a time-to-event outcome.

The schema in Fig. 3 summarizes the steps to implement the proposed approach.

## 4 Discussion

Fitting Cox *PH* models using traditional methods can result in the need for extensive computational resources and/or large blocks of computational time when analyzing time to event outcomes requiring fitting of voluminous *SNP* data such as typically encountered in the analysis of GWAS studies. Computing efficiency may be improved by substituting *CS adjusted* Poisson regression models and utilizing the *CS* residuals obtained from a single *PH* model containing only required predictor variables to be fitted to all models, omitting the *SNP*. Our proposed approach is to efficiently screen for *SNPs* of interest in GWAS studies which, in many cases, produce millions of candidate *SNPs* by utilizing the fact that *CS* residuals follow a unit exponential distribution. The approach yields effect estimates which closely approximate those of the *PH* models providing a computationally attractive alternative to such routine statistical analyses. The Bland–Altman plot comparing the estimates from Cox *PH* and Poisson models demonstrated excellent agreement and consistency between the two methods identifying which *SNPs* demonstrated promising association with the outcome. The gain in computational efficiency was on average > 60% for the 10 000 *SNPs* considered result in substantial time reduction after accounting for the extra processing time of cross-validation.

This approach has the advantage that if *PH* assumption is satisfied for all the *k* variables but not for some of the individual *SNPs*, this would, in general, not affect the Poisson model since the assumptions underpinning the Poisson distribution would usually be satisfied (i.e. occurrence of events is independent, only one event can occur in an instance of time, the events can be counted and the average rate of events can be calculated). While *SNP* screening using the deviance residuals gives remarkable time performance, the curvilinear profile of the standardized coefficients when compared to the *PH* model is concerning. This may be due in part to either the degree of censoring present in the outcome or, the frequency of cases in the *SNP* categories, or a combination of both. Isolation of which components drive this pattern in the deviance residuals is beyond the scope of this manuscript. Irrespective of the reasons for the curvilinear pattern, it sometimes may be preferable to trade off processing time gains and use the *CS adjusted* Poisson approach to achieve closer agreement with the Cox *PH* model estimates. This would be the case when analyses on a large number of *SNPs* is required and there are differing censoring patterns among the outcome variables. The lack of concordance of the deviance residuals with the result from the *PH* model appears to be a major limitation.

A different two-stage approach (Staley *et al.* 2017) is based on the fact that logistic regression is computationally more efficient than the *PH* model. A logistic regression to identify promising *SNPs* is first performed followed by *PH* modelling to explore associations using only the promising *SNPs*. Our approach has the advantage that the screening process adjusts for the *k* required variables in the *PH* model from which the CS residuals are derived. Since *k* predictor variables would still be required in any logistic model, we would expect the *CS adjusted* Poisson approach fitting just a single *SNP* to be more efficient.

The proposed approach has several advantages. First, when iteratively processing many variables via a looping procedure, the fact that each individual *SNP* analysis is relatively well defined as Poisson fitting provides assurance of greater numerical stability than a *PH* model requiring inversion of a (*k *+* *1) * (*k *+* *1) matrix formed via the score equations derived from the partial likelihood. Operations on sparse matrices can induce numerical instability which may substantially slow down analyses over and above the processing time component. The iterative component accounts for 30% of the processing time which is 70% of the time taken to fit the complete *CS adjusted* Poisson model. Numerical instability from fitting a Poisson model with a single explanatory variable can quickly be identified and appropriate remedial changes made (including omitting the *SNP* from further analysis). Secondly, once promising *SNPs* have been identified, they can be incorporated into multivariable Poisson regression models which can include interaction terms between *SNPs* and utilize variable selection methods (Miller 2002) to further refine classes of *SNPs* which correlate with each other, and have strong associations with the outcome. Provided *PH* assumptions for the Cox model are broadly satisfied, the issue of the degree of censoring present in the data reduces problems associated with the degree of censoring present in the data. Pragmatically, this feature is desirable if the impact of many *SNPs* being investigated on many outcomes with an automated fitting process. In such cases, detailed examination of the properties and assumptions of methods being used to identify important individual *SNPs* can be prohibitive.

Further advantages of our proposed approach include reducing the need for access to high-speed parallel processing computers with multiple CPU cores (often not readily available to many investigators) to perform the appropriate analyses particularly in studies examining fewer *SNPs*. Once a comprehensive *SNP* analysis has been performed, the *PH* model can be fitted using the variables of interest. Parameters can be estimated, the usual model diagnostics performed on a much smaller variable set and appropriate inferences made.

While we have outlined methods based on modelling the *CS* residuals through the Poisson/exponential distribution other approaches may also be considered. Performing a censored linear regression (Aitkin 1981) on *ln*(*r(i)_CS_*) may prove useful particularly if the effect of interest is the average time to event rather than the incidence density/hazard ratio. Here *ln*(*r(i)_CS_*) would be regressed on the *SNP* levels with the censoring status being that of the time to event outcome. Properties of such an approach still require evaluation. Other analytical approaches include the use of generalized linear models (GLM) and iterative hard thresholding treating all *SNPs* simultaneously (Chu *et al.* 2020). As *PH* models do not have the structure of exponential class models, it may be problematic to use GLM’s with time to event outcomes. A review of different strategies to detect interactions between *SNPs* is given in Cantor *et al.* (2010) although as the number of *SNPs* increases the computational efficiency is substantially diminished.

## 5 Conclusion

The approach of fitting individual *SNPs* to the *CS* residuals from a *PH* model comprising a common set of covariates using Poisson regression has been shown to produce estimates of *SNP* effects being numerically very close to those obtained from *PH* regression models containing, in turn, each *SNP* adjusted for this common set of covariates. Fitting of a univariate Poisson model however improves the computational and processing time efficiency. When tens of millions of *SNPs* need to be evaluated, the proposed method provides an effective screening strategy to identify promising *SNPs* and can facilitate higher dimensional analyses to identify familywise associations for those *SNPs* of interest. The approach is easily implemented on desktop computers and would not require access to high-performing computer systems with large memory allocations which may not be readily available to all investigators.

## Data Availability

As only a subset the FIELD data was only used to illustrate the performance of the proposed method, data availability would be considered on request to the authors.

## Appendix: Demonstration of the equivalence between the Cox *PH* and Poisson formulations

### Hypothetical dataset with two covariates *x*_1_ and *x*_2_: data layout

**Table:**

Original data					Data structure: augmented dataset for a Poisson model										
ID	x_1_	x_2_	Time	event	ID	*x*_1_	*x*_2_	Status	Time	Time 5	Time 11	Time 24	Time 27	Time 34	Time 45
1	0	40	5	1	1	0	40	1	5	1	0	0	0	0	0
2	1	23	11	1	2	1	23	0	5	1	0	0	0	0	0
3	1	37	15	0	3	1	37	0	5	1	0	0	0	0	0
4	0	67	16	0	4	0	67	0	5	1	0	0	0	0	0
5	1	44	24	1	5	1	44	0	5	1	0	0	0	0	0
6	0	48	27	1	6	0	48	0	5	1	0	0	0	0	0
7	1	29	28	0	7	1	29	0	5	1	0	0	0	0	0
8	0	59	30	0	8	0	59	0	5	1	0	0	0	0	0
9	1	18	34	1	9	1	18	0	5	1	0	0	0	0	0
10	1	55	40	0	10	1	55	0	5	1	0	0	0	0	0
11	0	18	45	1	11	0	18	0	5	1	0	0	0	0	0
					2	1	23	1	11	0	1	0	0	0	0
	
**Cox PH**		**Coefficient**		**HR**	3	1	37	0	11	0	1	0	0	0	0
	
*x*_1_		0.0566		1.0583	4	0	67	0	11	0	1	0	0	0	0
*x*_2_		−0.0099		0.9902	5	1	44	0	11	0	1	0	0	0	0
					6	0	48	0	11	0	1	0	0	0	0
	
**Poisson**		**Coefficient**		**IDR**	7	1	29	0	11	0	1	0	0	0	0
	
Intercept		−2.0500		0.1287	8	0	59	0	11	0	1	0	0	0	0
*x*_1_		0.0566		1.0583	9	1	18	0	11	0	1	0	0	0	0
*x*_2_		−0.0099		0.9902	10	1	55	0	11	0	1	0	0	0	0
Time 11		0.0907		1.0950	11	0	18	0	11	0	1	0	0	0	0
Time 24		0.4406		1.5536	5	1	44	1	24	0	0	1	0	0	0
Time 27		0.588		1.8004	6	0	48	0	24	0	0	1	0	0	0
Time 34		1.2006		3.3220	7	1	29	0	24	0	0	1	0	0	0
Time 45		2.2276		9.2777	8	0	59	0	24	0	0	1	0	0	0
	
IDR: Incidence density ratio					9	1	18	0	24	0	0	1	0	0	0
					10	1	55	0	24	0	0	1	0	0	0
					11	0	18	0	24	0	0	1	0	0	0
					6	0	48	1	27	0	0	0	1	0	0
					7	1	29	0	27	0	0	0	1	0	0
					8	0	59	0	27	0	0	0	1	0	0
					9	1	18	0	27	0	0	0	1	0	0
					10	1	55	0	27	0	0	0	1	0	0
					11	0	18	0	27	0	0	0	1	0	0
					9	1	18	1	34	0	0	0	0	1	0
					10	1	55	0	34	0	0	0	0	1	0
					11	0	18	0	34	0	0	0	0	1	0
					11	0	18	1	45	0	0	0	0	0	1

### Calculation of the cumulative baseline hazard from the Poisson formulation

**Table:**

Time period	Coefficient	log(hazard)	Hazard	Cumulative hazard
0			0	0
5	−2.0500	−2.0500	0.1287	0.1287
11	0.0907	−1.9593	0.1410	0.2697
15			0	0.2697
16			0	0.2697
24	0.4406	−1.6094	0.2000	0.4697
27	0.5880	−1.4620	0.2318^a^	0.7015
28			0	0.7015
30			0	0.7015
34	1.2006	−0.8494	0.4277	1.1292
40			0	1.1292
45	2.2276	0.1776	1.1943	2.3236

a *e* ^(−2.0500 + 0.5880)^ = 0.2318.

### Cox–Snell residuals

**Table:**

ID	*x*_1_	*x*_2_	Time	Event	$e^{\beta_{1}x_{1}+\beta_{2}x_{2}}$	Cox–Snell residuals *r(i)_CS_*	
						Poisson	Cox
1	0	40	5	1	0.6738	0.0867	0.0867
2	1	23	11	1	0.8434	0.2275	0.2275
3	1	37	15	0	0.7345	0.1981^a^	0.1981
4	0	67	16	0	0.5162	0.1392	0.1392
5	1	44	24	1	0.6855	0.322	0.322
6	0	48	27	1	0.6227	0.4368	0.4368
7	1	29	28	0	0.7949	0.5576	0.5576
8	0	59	30	0	0.5586	0.3919	0.3919
9	1	18	34	1	0.886	1.0005	1.0005
10	1	55	40	0	0.615	0.6944	0.6944
11	0	18	45	1	0.8372	1.9454	1.9454

a 0.7345*0.2697 = 0.l981.
